# Anterior Elbow Dislocation Associated With Medial Epicondyle and Humeral Condyle Fractures in a Pediatric Patient: A Report of a Rare Case

**DOI:** 10.7759/cureus.113769

**Published:** 2026-08-01

**Authors:** Andrés Felipe Muñoz Leiva, Juan Pablo Aguirre Echeverry, Camilo Andres Padilla Martinez, Dolores Cantu Fernandez, Leobardo Guerrero Beltran

**Affiliations:** 1 Orthopedics and Traumatology, Hospital Juárez de México, Mexico City, MEX; 2 Faculty of Medicine, Universidad Nacional Autónoma de México, Mexico City, MEX

**Keywords:** anterior dislocation, elbow dislocation, kirschner wires, medial epicondyle fracture, pediatric trauma

## Abstract

We present the case of an 11-year-old boy who sustained an anterior elbow dislocation associated with fractures of the medial epicondyle and humeral condyle following a fall from playground equipment. Anterior elbow dislocation is an exceptionally rare injury in the pediatric population, and its association with concomitant articular fractures further increases its clinical complexity. Early diagnosis and prompt surgical management were essential to prevent anatomical and functional sequelae. The patient underwent closed reduction followed by open reduction and internal fixation with Kirschner wires due to the presence of displaced osseous fragments. At the four-month follow-up, the patient achieved complete elbow range of motion without pain or functional limitation. This case highlights the importance of maintaining a high index of clinical suspicion for associated fractures in pediatric anterior elbow dislocations and underscores the need for an individualized, timely treatment approach to optimize outcomes in this rare injury pattern.

## Introduction

Elbow dislocation is the most common dislocation of the upper extremity in the pediatric population and frequently occurs in association with osseous and ligamentous injuries. Elbow dislocations account for 3-6% of pediatric elbow injuries [1].

The elbow joint is a complex articulation comprising three distinct joints: the ulnohumeral, the radiocapitellar, and the proximal radioulnar articulations. Movements at the elbow include flexion and extension, as well as forearm pronation and supination. Primary stabilizers of the elbow include the ulnohumeral bony congruence, the anterior band of the medial collateral ligament, and the lateral collateral ligament complex; secondary stabilizers encompass the radiocapitellar joint, the articular capsule, the common flexor and extensor muscle origins, and the dynamic muscular stabilizers [2,3].

Elbow dislocations are classified according to the direction of displacement as posterior, anterior, lateral, or medial. Posterior dislocation, typically resulting from a fall on an outstretched hand, is by far the most frequent variant. Anterior dislocation, in contrast, is exceptionally rare and most commonly results from a direct impact to the posterior aspect of a flexed elbow [4,5]. Approximately 64% of pediatric elbow dislocations are associated with concomitant fractures, most frequently involving the lateral condyle or the medial epicondyle [6,7].

We present the case of an 11-year-old boy who sustained an anterior elbow dislocation with associated fractures of the medial epicondyle and humeral condyle following a fall from playground equipment, with the aims of contributing to the limited literature on this rare injury pattern and discussing the diagnostic and therapeutic approach employed.

## Case presentation

An 11-year-old boy with no relevant medical history presented to the emergency department following an accidental fall from playground equipment. According to the accompanying family member, the child sustained a direct impact to the right elbow upon landing. On physical examination, the patient presented with severe pain and marked deformity of the right elbow and maintained the forearm in a fixed position of neutral pronation-supination, supported against the thorax with the contralateral hand. Neurovascular assessment of the distal upper extremity was intact.

**Figure 1:**
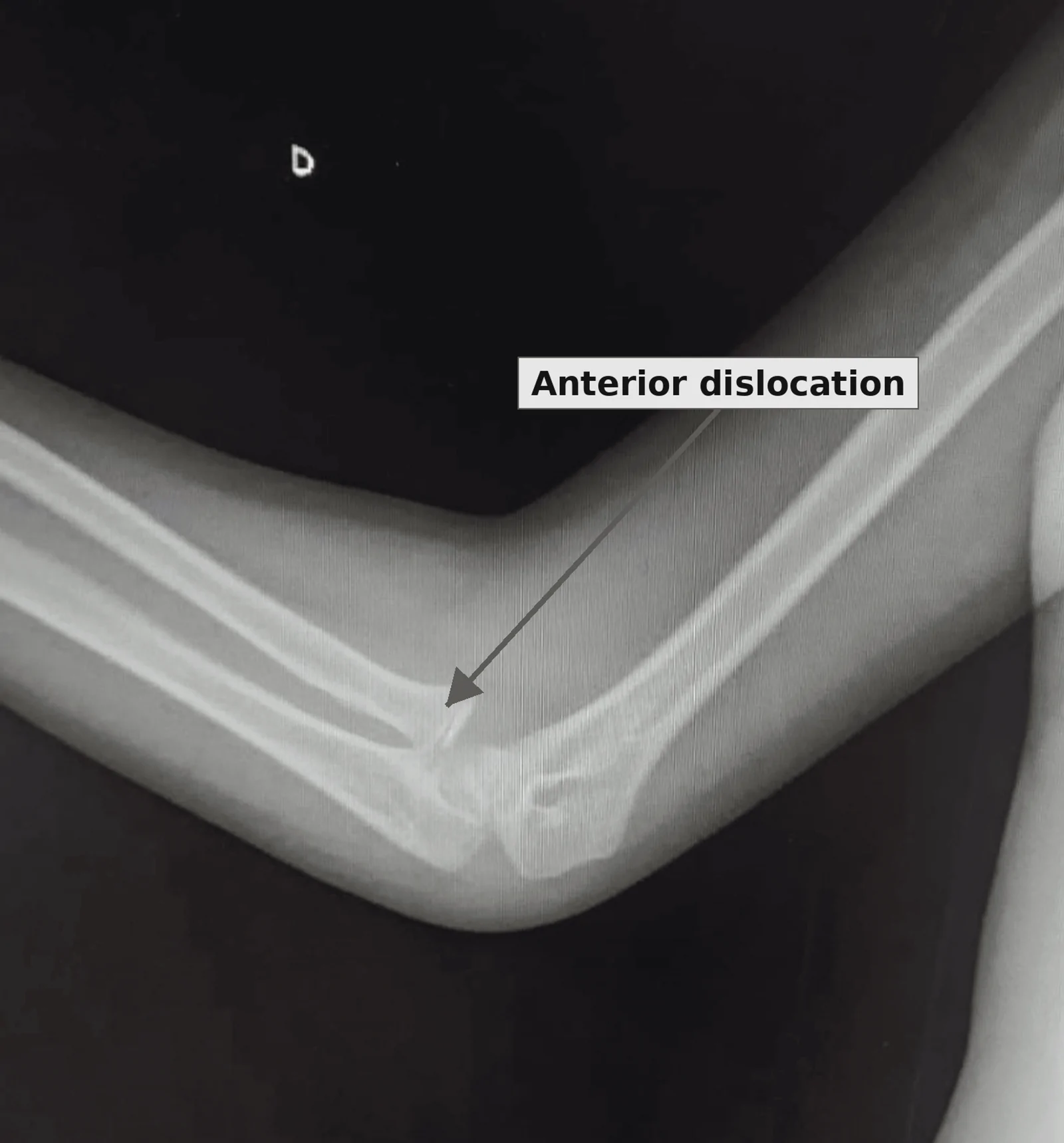
Lateral radiograph of the right elbow demonstrating anterior dislocation of the radius and ulna prior to reduction.

**Figure 2:**
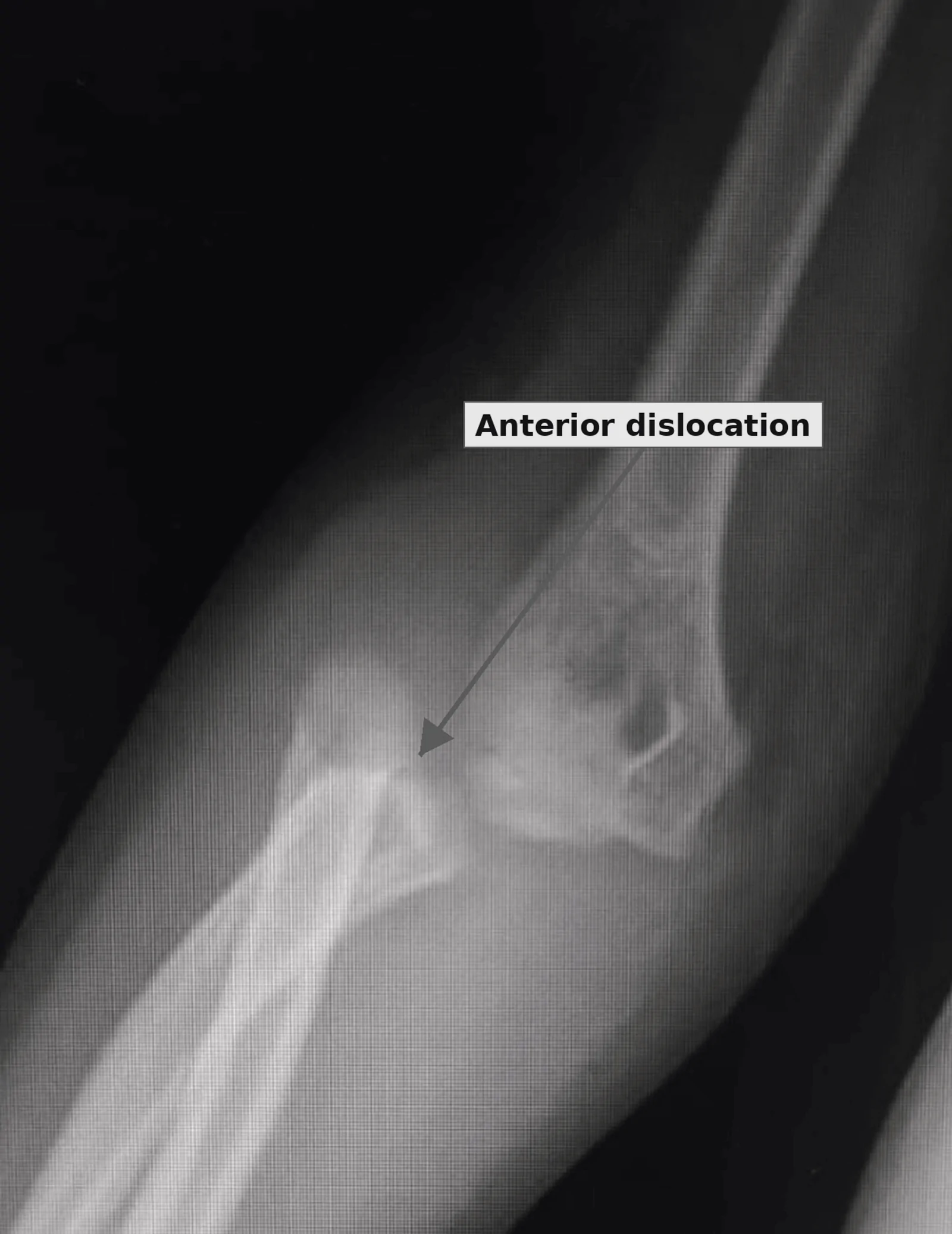
Anteroposterior radiograph of the right elbow confirming anterior dislocation with apparent associated osseous fragments involving the medial epicondyle and humeral condyle prior to reduction.

Plain radiographs of the right elbow in two projections were obtained and were sufficient to establish the diagnosis of anterior dislocation of the elbow joint, demonstrating anterior displacement of the radius and ulna relative to the humerus, with apparent associated osseous fragments (Figure 1, Figure 2). A prominent olecranon was noted posteriorly on physical examination, consistent with the radiographic findings.

Closed reduction was performed in the diagnostic imaging area under combined procedural sedoanalgesia with intravenous ketamine and midazolam, with fluoroscopic guidance used to confirm real-time articular alignment during the maneuver. The reduction technique consisted of longitudinal traction along the axis of the forearm, while an assistant stabilized the humerus, followed by gradual flexion of the elbow combined with a posteriorly and distally directed force on the proximal forearm. A palpable and audible "clunk" was noted during the maneuver, after which the posterior deformity resolved and articular congruence was restored. Elbow range of motion was assessed under sedation to evaluate post-reduction stability. The patient reported immediate improvement in pain following the procedure.

**Figure 3:**
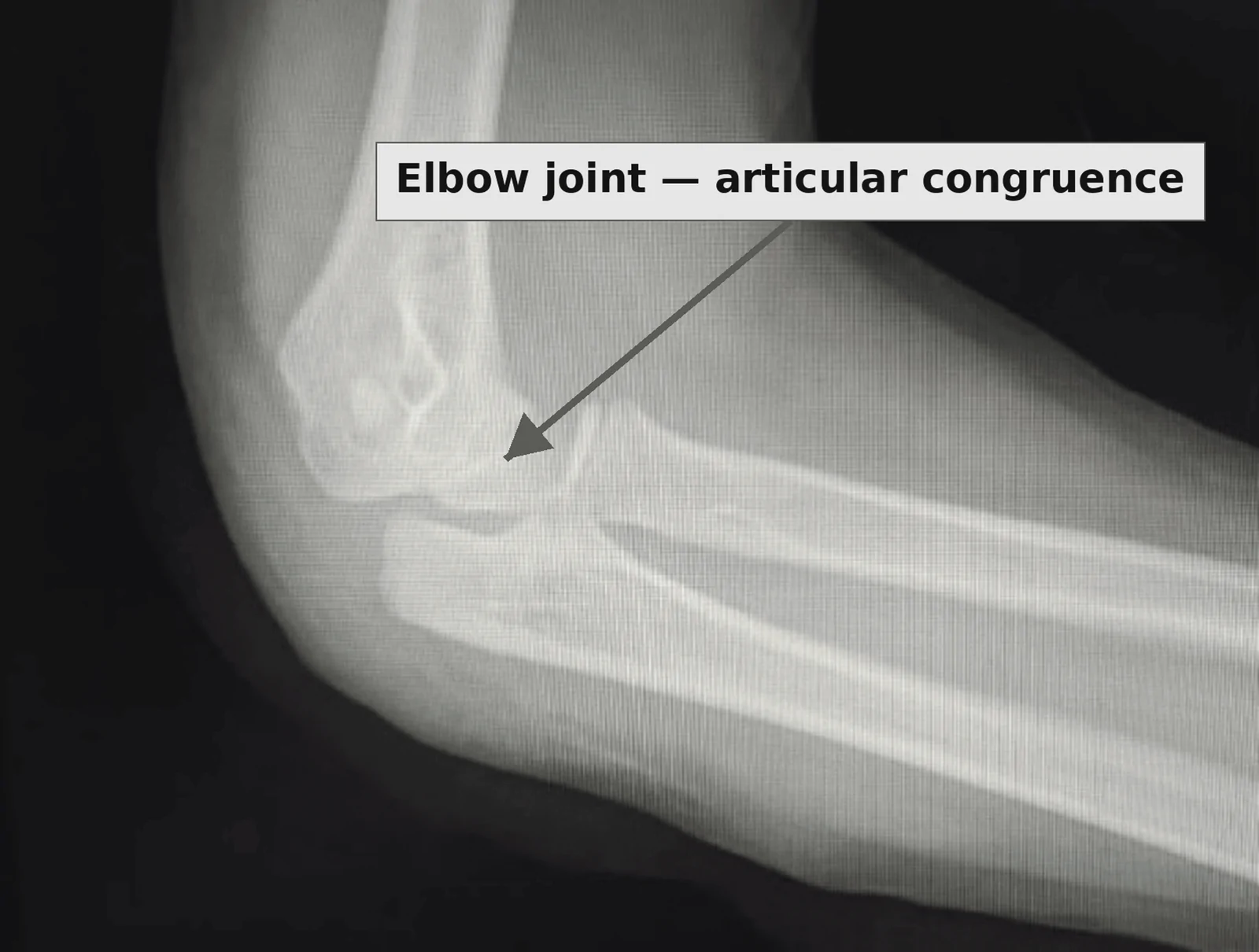
Lateral radiograph of the right elbow following closed reduction demonstrating satisfactory restoration of articular congruence between the proximal ulna and the humeral condyle.

Post-reduction radiographs confirmed satisfactory articular congruence between the proximal ulna and the humeral condyle (Figure 3). However, imaging also revealed displaced fracture fragments involving the humeral condyle and medial epicondyle, precluding conservative management alone. The elbow was temporarily immobilized with a posterior splint at 90° of flexion and neutral forearm rotation pending surgical planning.

**Figure 4:**
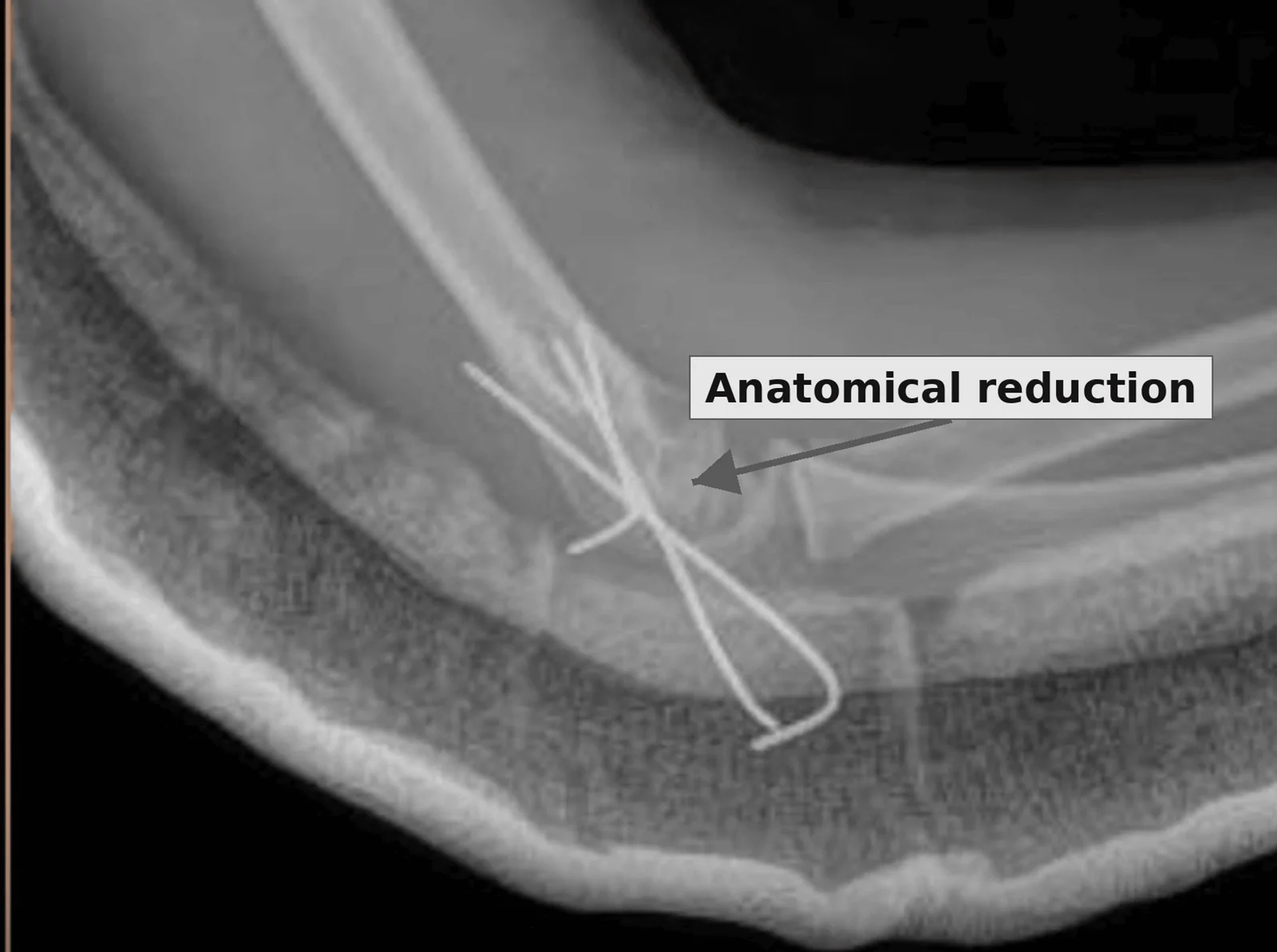
Lateral radiograph of the right elbow following open reduction and internal fixation demonstrating anatomical reduction secured with divergent 1.6 mm Kirschner wires.

**Figure 5:**
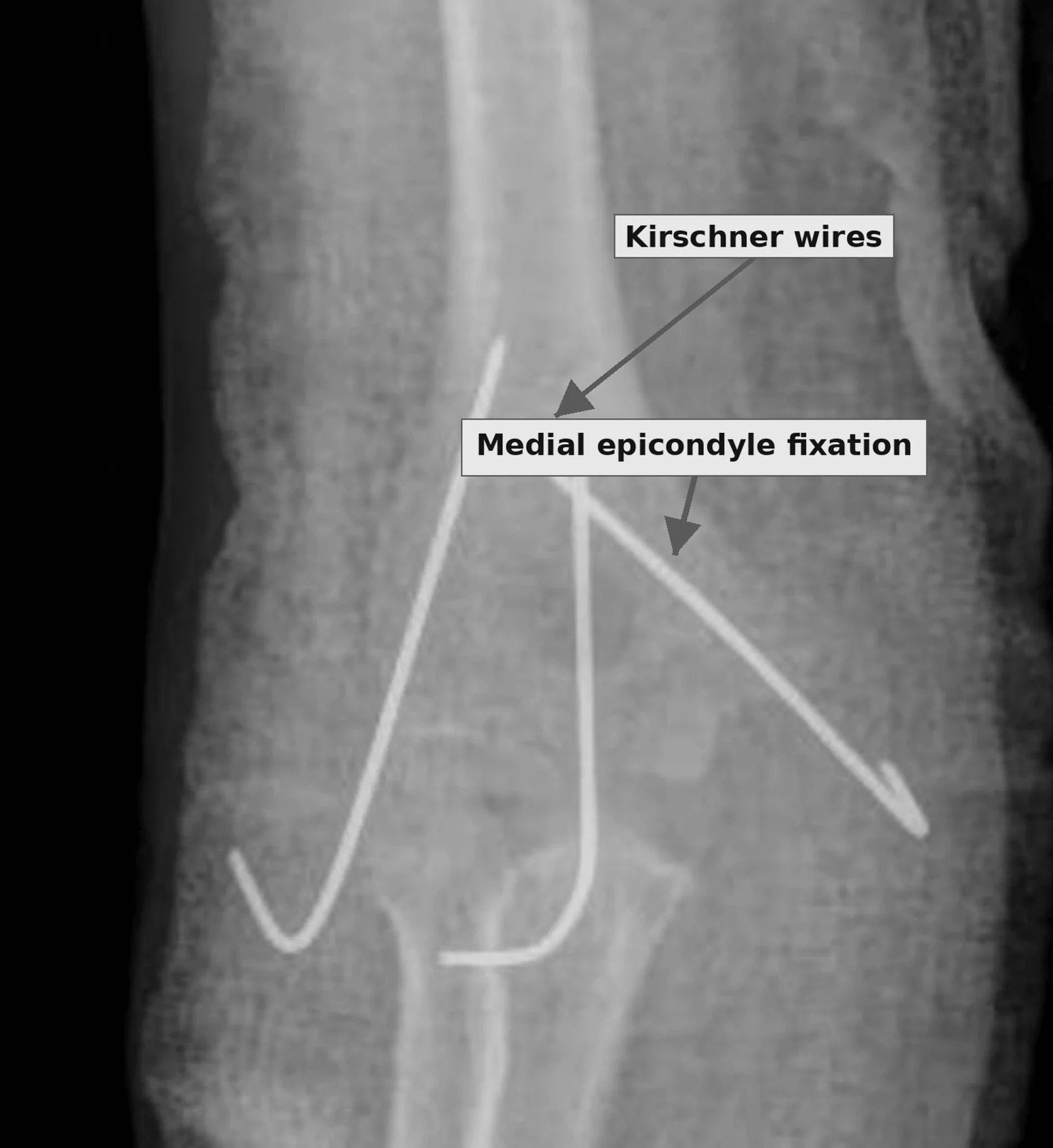
Anteroposterior radiograph of the right elbow following open reduction and internal fixation confirming Kirschner wire placement and anatomical fixation of the medial epicondyle and humeral condyle fragments.

The patient was admitted and subsequently underwent open reduction and internal fixation via a posterolateral approach, with an operative time of approximately one hour. The ulnar nerve was protected through medial referencing and isolation with a Penrose drain throughout the procedure. Intraoperative exploration confirmed a medial epicondyle fracture together with a humeral condyle fracture with associated articular incongruence, and stress testing ruled out associated ligamentous injury. One Kirschner wire was used to fix the medial epicondyle fragment, and two divergent 1.6 mm Kirschner wires were used to fix the humeral condyle fragment (Figure 4, Figure 5). The reduction was protected postoperatively with a plaster cast at 90° of elbow flexion. At approximately six weeks postoperatively, the cast and Kirschner wires were removed, and progressive range-of-motion rehabilitation was initiated immediately. At the two-month follow-up, the patient demonstrated favorable clinical evolution. At the four-month follow-up, the patient achieved complete elbow range of motion without pain and with no functional limitation.

## Discussion

Anterior elbow dislocation is an exceptionally uncommon injury, particularly in the pediatric population, with only a limited number of cases documented in the medical literature since its first description following a direct blow to the posterior olecranon [6]. In contrast to posterior dislocation, which typically results from a fall on an outstretched hand and constitutes the overwhelming majority of elbow dislocations, anterior dislocation arises most commonly from a direct impact to the posterior aspect of a flexed elbow, as observed in the present case [4,5]. The rarity of this injury pattern, combined with its potential for associated osseous and ligamentous damage, makes early recognition and prompt management essential to prevent short- and long-term functional sequelae.

The stability of the elbow joint depends on a coordinated interplay between primary and secondary stabilizers. Primary stabilizers include ulnohumeral bony congruence, the anterior band of the medial collateral ligament, and the lateral collateral ligament complex. Secondary stabilizers encompass the radiocapitellar joint, the articular capsule, the common flexor and extensor muscle origins, and the dynamic muscular stabilizers. Disruption of these structures as occurs in anterior dislocation predisposes the joint to instability and increases the likelihood of associated fractures. Indeed, approximately 64% of pediatric elbow dislocations present with concomitant fractures, most frequently involving the lateral condyle or the medial epicondyle [7]. The simultaneous presence of medial epicondyle and humeral condyle fractures in this case reflects the significant energy transfer sustained during the injury and underscores the importance of thorough post-reduction imaging to identify all associated osseous lesions.

Comparison with reported cases

The present case shares several features with the limited published series on pediatric anterior elbow dislocation. Jlidi et al. reported an anterior trans-olecranial dislocation in a child, also resulting from direct posterior impact, managed successfully with closed reduction followed by surgical fixation of associated fractures [5]. Similarly, Dimantha et al. described a pediatric anterior dislocation arising from an atypical mechanism, highlighting the variability in injury patterns and the importance of individualized management [4]. Alotaibi and Alfaya reported an isolated anterior dislocation in an adolescent without associated fractures, managed conservatively with excellent outcomes [6], in contrast to our case where the presence of displaced fractures necessitated open surgical intervention. Collectively, these reports suggest that while the mechanism and degree of associated injury may vary, early diagnosis and prompt reduction remain the cornerstone of management regardless of the specific presentation. Early functional mobilization principles, initially described in the context of non-operative treatment of simple elbow dislocations, have also been extrapolated to guide rehabilitation following surgically fixed pediatric elbow fracture-dislocations, as applied in the present case.

Differential diagnosis

The differential diagnosis of anterior elbow dislocation in children includes posterior elbow dislocation, Monteggia fracture-dislocation, and supracondylar humeral fracture. Posterior dislocation, the most common variant, is readily distinguished by the direction of displacement on lateral radiographs, with posterior positioning of the radius and ulna relative to the humerus. Monteggia fracture-dislocation involves an ulnar shaft fracture with associated radiocapitellar dislocation and should be systematically excluded in all pediatric elbow injuries by verifying radiocapitellar alignment on all radiographic projections. Supracondylar fractures may mimic dislocation clinically due to significant periarticular swelling and deformity, but are distinguished by the presence of a discrete fracture line through the distal humerus without complete joint dislocation. In the present case, the characteristic anterior displacement of both the radius and ulna on lateral radiography, combined with the absence of an ulnar shaft fracture, confirmed the diagnosis of anterior dislocation with associated intra-articular fractures.

Diagnosis

Diagnosis of anterior elbow dislocation is primarily clinical, supported by plain radiography as the initial imaging modality. The characteristic physical examination findings, a prominent posterior olecranon, fixed forearm positioning, and marked periarticular deformity, combined with lateral radiographic views demonstrating anterior displacement of the radius and ulna relative to the humerus, are generally sufficient to establish the diagnosis without the need for advanced imaging in the acute setting. Computed tomography may be considered in cases where plain radiographs are inconclusive or where the extent of associated osseous injury requires further characterization prior to surgical planning.

Treatment

The management of pediatric elbow dislocations is primarily guided by the degree of displacement, the presence of associated fractures, and the stability achieved following reduction. The majority of cases are amenable to closed reduction under adequate analgesia or sedation, followed by immobilization [8,9]. Closed reduction must be performed with particular care in the pediatric population to protect the physeal growth plates, avoid iatrogenic nerve entrapment, and prevent additional fractures during the maneuver. Indications for open reduction and surgical fixation include failure of closed reduction, the presence of displaced fractures, persistent post-reduction instability, interposed soft tissue, or delayed presentation [9,10].

In the present case, while closed reduction was successfully achieved, post-reduction imaging identified displaced fracture fragments involving both the humeral condyle and the medial epicondyle, necessitating open reduction and internal fixation with divergent 1.6 mm Kirschner wires. This approach allowed the anatomical restoration of the articular surfaces and provided sufficient stability for early rehabilitation. The timing of Kirschner wire removal at six weeks and the immediate initiation of progressive range-of-motion exercises reflect current recommendations for minimizing the risk of post-traumatic elbow stiffness, which represents one of the most frequent complications following elbow dislocation in children [11].

Rehabilitation followed a structured, phase-based protocol emphasizing active over passive mobilization, consistent with pediatric-specific recommendations to minimize the risk of stiffness and heterotopic ossification. Initial immobilization was maintained until hardware removal, followed by active or active-assisted range-of-motion exercises, progressive strengthening once full mobility was regained, and a graduated return to normal activity guided by functional and radiographic criteria rather than a fixed timeline. The choice of Kirschner wire fixation over cannulated screws was guided primarily by the patient's age and the immaturity of the epiphyseal ossification, which limited adequate purchase for screw fixation; this approach is supported by recent literature indicating comparable long-term functional outcomes between the two methods in pediatric patients, with Kirschner wires offering the additional advantage of straightforward outpatient removal without a second surgical procedure.

## Conclusions

Anterior elbow dislocation in the pediatric population represents an exceptionally rare injury pattern that demands a high index of clinical suspicion and a systematic diagnostic approach. The present case illustrates that anterior dislocation may occur in association with displaced intra-articular fractures, significantly increasing the complexity of management and the risk of functional sequelae if not promptly identified and treated. The successful outcome achieved in this patient, complete elbow range of motion without pain or functional limitation at the four-month follow-up, underscores the importance of early diagnosis, appropriate reduction technique, and timely surgical intervention when indicated. When closed reduction alone is insufficient due to associated displaced fractures, open reduction and internal fixation with Kirschner wires may be an effective option in the pediatric population, provided it is followed by early functional rehabilitation.

Clinicians encountering pediatric elbow trauma should maintain awareness of anterior dislocation as a possible albeit uncommon injury pattern, particularly when the mechanism involves a direct posterior impact to a flexed elbow. Post-reduction imaging is essential in all cases to identify concomitant osseous lesions that may alter the treatment plan. Early mobilization following adequate stabilization remains the cornerstone of functional recovery and should be prioritized to minimize the risk of post-traumatic stiffness.
